# A Method for Extracting Sedimentary Outcrops from UAV Oblique Photogrammetry Point Clouds

**DOI:** 10.3390/s26123946

**Published:** 2026-06-21

**Authors:** Chufan Ren, Chaodong Wu, Yanan Zhang, Cong Lin, Xinyue Niu, Yanan Chu

**Affiliations:** Key Laboratory of Orogenic Belts and Crustal Evolution, Ministry of Education, School of Earth and Space Sciences, Peking University, Beijing 100871, China; cf_ren@126.com (C.R.); yn_zhang@stu.pku.edu.cn (Y.Z.); 2101110669@stu.pku.edu.cn (C.L.); 2501110629@stu.pku.edu.cn (X.N.); chuyn@stu.pku.edu.cn (Y.C.)

**Keywords:** sedimentary outcrops, point-cloud semantic segmentation, cross-scale self-attention, stratigraphic attitude, UAV oblique photogrammetry, Qingshuihe Formation

## Abstract

Point-cloud analysis of sedimentary outcrops using Unmanned Aerial Vehicle (UAV) oblique photogrammetry is a crucial approach to sedimentary system characterization, stratigraphic correlation, and petroleum exploration analog studies. In large-scale field settings, however, outcrops are often scattered and fragmented, vegetation and soil cover is extensive, and class imbalance is pronounced. Manual interpretation is labor-intensive, while existing clustering algorithms, conventional machine learning methods, and general-purpose point-cloud segmentation networks struggle to simultaneously ensure geometric fidelity, rare-class recognition, and multi-scale feature integration. To address these challenges, we propose a method for extracting sedimentary outcrop point clouds from field surface point clouds using a UAV oblique photogrammetry acquisition strategy. The core segmentation module of the method, sedimentary cross-scale self-attention network (SedCSA-Net), is an enhanced version of PointNet++ that integrates collaborative improvements across four dimensions: data augmentation, sampling strategy, feature encoding, and loss optimization. Taking the Cretaceous Qingshuihe Formation in the Louzhuangzi area of the southern Junggar Basin as a case study, our experimental results indicate that SedCSA-Net overcomes the natural variability of UAV oblique photogrammetry point clouds—such as shadows, voids, and uneven density—achieving a mean Intersection over Union(mIoU) of 89.51% and an Overall Accuracy(OA) of 96.08%, with an outcrop-class Intersection over Union(IoU) of 86.90%. Attitude measurements derived from segmentation results deviate by less than 3° from manually annotated references, demonstrating that the proposed framework provides an end-to-end, generalizable approach for intelligent segmentation, geometric reconstruction, and attitude extraction of large-scale sedimentary outcrop point clouds.

## 1. Introduction

A sedimentary outcrop is a visible cross-section or outcrop body of naturally exposed sedimentary strata and their associated geological structures at the Earth’s surface [1]. Sedimentary outcrops preserve key information on stratigraphic sequences, sedimentary structures, palaeontological traces, and the evolution of depositional environments and are a core geological archive for geologists to analyze sedimentary processes, reconstruct palaeogeographic configurations, and conduct reservoir-correlation studies [2,3,4].

With the rapid proliferation of Unmanned Aerial Vehicle (UAV) platforms and Structure-from-Motion(SfM) technology [5], three-dimensional digital reconstruction of large-scale geological landforms from an aerial perspective has become feasible. Using UAVs equipped with multi-lens oblique-camera systems, researchers can acquire high-density image sequences of kilometer-scale outcrops in a single field campaign and, through SfM algorithms and Multi-View Stereo(MVS) matching, generate dense three-dimensional point clouds carrying both spatial coordinates and color information [4]. Quantitatively extracting sedimentary outcrops from large-scale field surface point clouds provides substantial value for sedimentological studies, such as stratigraphic analysis, depositional-environment reconstruction, and reservoir characterization [1,3,4].

Nevertheless, extracting outcrops from large-scale field surface point clouds poses many challenges. First, outcrop morphology is inherently complex and discontinuous: structural deformation, differential weathering, and partial vegetation cover produce irregular outcrop geometries with blurred boundaries that grade gradually into soil and biological cover, lacking consistent geometric discontinuities for segmentation. Second, point-cloud acquisition methods in existing studies are not suited to kilometer-scale surveys. Third, kilometer-scale surveys involve a trade-off between resolution and data volume, requiring adaptation to lower-resolution data. Fourth, field surface classes are highly imbalanced.

To meet these challenges, we propose a sedimentary outcrop extraction method oriented toward kilometer-scale field point clouds. The method integrates UAV oblique photogrammetry point-cloud acquisition and cleaning, geological-prior feature enhancement, class-balanced sampling, cross-scale self-attention feature encoding, hybrid-loss optimization, and segmentation-based bedding-surface fitting and attitude measurement into a single end-to-end pipeline.

Geological scales are hierarchical (lamina - bed - bedset), which corresponds naturally to the hierarchical local aggregation of PointNet++ [6,7]. In addition, PointNet++ has the advantages of a simple structure, small parameter scale, and high computational efficiency, which has obvious practical advantages in the working scenario of limited hardware resources and unstable networks, such as field geological surveys [6,7]. Accordingly, the core segmentation module of our method follows the hierarchical feature-extraction philosophy of PointNet++ and introduces four coordinated improvements, yielding SedCSA-Net (sedimentary cross-scale self-attention network): (1) geological-prior enhanced inputs composed of normal vectors, curvature, roughness, and color gradients [8]; (2) a weighted random-sampling strategy keyed on the point-count proportion of rare classes [9]; (3) a cross-scale self-attention (CSA) module built on top of Multi-Scale Grouping (MSG) [7,10,11]; and (4) a loss function combining Focal–Tversky loss [12] and Lovász–Softmax loss [13] in a weighted manner. While retaining the computational efficiency of PointNet++, the framework provides a systematic adaptation to large-scale, imbalanced, multi-morphology sedimentary outcrop point clouds.

The study area is the Cretaceous Qingshuihe Formation in the Louzhuangzi area on the southern margin of the Junggar Basin [14], a region with intense structural deformation [15,16], where outcrops display diverse morphologies and are obscured to varying degrees by vegetation and soil [17,18]. In this area, we construct a kilometer-scale sedimentary outcrop point-cloud dataset and evaluate the proposed framework under a strict spatial extrapolation partition.

The main contributions of this paper are summarized as follows:

(1) We construct a large-scale sedimentary outcrop point-cloud dataset acquired under real field conditions using UAV oblique photogrammetry. The dataset explicitly preserves realistic acquisition characteristics—including shadow-induced voids, point-density heterogeneity, and SfM reconstruction artifacts—thereby providing a benchmark that faithfully represents actual deployment conditions rather than idealized survey geometries.
(2) We propose SedCSA-Net, a domain-knowledge-driven segmentation architecture that explicitly encodes three categories of sedimentary geological priors as learnable input features and combines a cross-scale attention mechanism with a class-imbalance-aware training strategy, designed specifically for large-scale UAV oblique photogrammetry outcrop point clouds.
(3) We validate the proposed framework on the constructed dataset using a spatial extrapolation partition scheme that tests the model’s ability to generalize to geographically unseen outcrop regions. The results show that the framework outperforms existing baselines and enables automatic bedding-attitude measurement with deviations of less than 3° from field reference values, thereby establishing a complete workflow from raw UAV point-cloud acquisition to quantitative geological characterization.

## 2. Related Work

Existing research on sedimentary outcrop point-cloud segmentation can be categorized by data source and input modality into Light Detection and Ranging ( LiDAR)-based methods and UAV oblique-photogrammetry-based methods.

### 2.1. LiDAR-Based Methods for Sedimentary Outcrop Interpretation

LiDAR is an active remote sensing technique that directly acquires high-precision three-dimensional spatial coordinates and reflectance intensity by emitting laser pulses and recording return signals. It has become a vital data source for quantitative sedimentary outcrop analysis. Terrestrial Laser Scanning(TLS), in particular, offers millimeter-level geometric accuracy and single- or multi-wavelength reflectance information, and dominated early digital outcrop studies [4,19].

Regarding TLS intensity utilization, early work relied mainly on reflectance differences for lithological discrimination [20,21,22]. Franceschi et al. [23] first distinguished marls from limestones using single-wavelength TLS intensity data, verifying the feasibility of intensity features. Hartzell et al. [24] went beyond the single-wavelength limitation and explored the application of multispectral LiDAR in virtual-outcrop geology, demonstrating that combining reflectances at different wavelengths can substantially improve lithological discrimination.

However, relying solely on intensity information is prone to confusion when the geological background is complex, so subsequent studies generally turned to the joint use of geometry and intensity: Weidner et al. [25] applied Random Forests to TLS point clouds for multi-class segmentation into vegetation, soil, debris, and outcrop categories. Liu et al. [20] extended this by integrating geometric features, reflectance, and scan attributes into a multi-dimensional feature vector for lithological classification using Random Forests. Liu et al. [22] proposed a channel-attention-based lithology segmentation method using multi-feature voxel values from outcrops, achieving robust discrimination between crystalline and clastic rocks through channel-wise feature reweighting. The common strength of this stage of methods is their strong feature interpretability, while their weakness is that feature engineering depends heavily on manual expertise and generalization is limited.

Recent TLS-based methods have further advanced multi-scale feature extraction and data sparsification, reflecting a trend from hand-crafted features toward automated structured feature learning. Gan et al. [21] introduced a Stratigraphically Constrained Continuous Clustering(SCCC) framework that combines dynamic density-threshold hierarchical clustering with block-level geometric–spectral feature aggregation, refining fine-grained lithological boundaries and achieving an Overall Accuracy(OA) of 94.64% on the Qingshuihe Formation dataset. Liu et al. [22] developed the MC-H-Geo multi-scale contextual hierarchical framework, combining voxel anchor construction, cross-scale differential features, and gated mixture-of-experts classifiers to improve TLS outcrop lithology classification. Duan et al. [26] proposed a hierarchical multi-feature framework based on Feature-Preserved Compressive Sampling(FPCS), which compresses point clouds via graph signal processing while preserving key textural and geometric features, substantially reducing data volume without sacrificing accuracy. Compared with the preceding stage, these methods show clear gains in both accuracy and efficiency and demonstrate stronger adaptability in difficult scenarios such as complex interbed transitions and blurred boundaries; however, the high cost of training data and annotation remains a bottleneck limiting their large-scale application.

Overall, by virtue of millimeter-level precision and rich multi-dimensional features, TLS methods achieve high-quality, fine-grained lithological and lithofacies discrimination on local cross-sections at the meter-to-ten-meter scale, representing the current state of the art for detailed local-outcrop interpretation. TLS equipment is costly, and deployment is challenging, making single-session acquisition of kilometer-scale scenes impractical. Additionally, these methods rely heavily on handcrafted feature engineering, which typically requires re-tuning for new study areas. This fundamental scale bottleneck has prompted researchers to seek alternative means that balance coverage and interpretation accuracy.

### 2.2. UAV Oblique-Photogrammetry-Based Methods for Sedimentary Outcrop Interpretation

The application of UAV platforms and SfM algorithms has markedly expanded the observation scale of sedimentary outcrop digitization. Constrained by station layout and field-of-view occlusion, traditional TLS can usually obtain only meter-scale local cross-sections; UAV oblique photogrammetry, by mounting a multi-lens oblique camera on a UAV platform, can synchronously acquire imagery of ground features from multiple viewpoints and cover broad kilometer-scale areas in a single flight. The technique adapts well to complex terrain, and multi-angle imagery effectively reduces occlusion blind spots in regions such as steep slopes and cliff faces, making it particularly suitable for efficient data acquisition of large-area outcrops in field geological surveys. Compared with methods such as TLS, oblique photogrammetry does not require laying out numerous stations in the field; it is simple and highly mobile to operate on site and can rapidly acquire three-dimensional data over an entire outcrop corridor at relatively low cost, providing a practical technical pathway for subsequent large-scale digital-outcrop modeling.

Current geological interpretation work based on UAV oblique photogrammetry point clouds follows two technical routes.

The first route is based on two-dimensional image segmentation with three-dimensional projection. This route directly leverages mature two-dimensional deep-learning frameworks, using high-resolution texture imagery as the primary information carrier. Researchers have combined Local Binary Pattern(LBP) texture features with Convolutional Neural Network(CNN) image segmentation to achieve multi-class lithofacies segmentation of mixed carbonate–clastic outcrops and to generate three-dimensional lithofacies point clouds [27]; others have proposed an “automatically segment image–reproject to 3D” workflow, using U-Net to segment turbidite-outcrop imagery before reprojection, effectively reducing the manual-annotation workload [28]. The advantage of this route is that it can reuse a large number of pre-trained two-dimensional vision models and fully exploit texture information; its drawback is that the two-dimensional projection process introduces occlusion errors and geometric distortion, label conflicts readily arise when fusing multi-angle imagery, and three-dimensional spatial consistency is difficult to guarantee.

The second route processes point-cloud data directly in three-dimensional space [29,30]. Researchers have voxelized point clouds and fed them into octree-based 3D convolutional networks, combined with spatial parameters to achieve stratigraphic segmentation, and further extended this to a spatial case-based reasoning model for stratigraphic identification; others have identified clastic-rock outcrop lithology on multimodal UAV imagery and point clouds or clustered outcrops in CIELAB color space to separate fractures from the background [31]. This route has good spatial consistency and preserves complete three-dimensional structural information, but as point-cloud resolution decreases from centimeter to decimeter level, micro-textures of rock layers become blurred, the network struggles to extract sufficiently discriminative features from sparse point clouds, and the computational cost is usually markedly higher than that of two-dimensional methods.

The two routes have different emphases: the former is dominated by texture information, and the latter is centered on geometric structure. How to effectively exploit high-resolution texture information while maintaining three-dimensional spatial consistency remains a key challenge for oblique photogrammetry point-cloud segmentation. On the other hand, inherent problems of oblique photogrammetry point clouds—point density varying with flight height, color distortion caused by illumination/shadow, and SfM reconstruction errors accumulating in steep-wall regions—impose higher robustness requirements on segmentation models: the model must maintain stable feature-extraction capability under non-uniform point density and tolerate a degree of color noise and geometric voids so as to reliably perform semantic segmentation even on real field point clouds that have not undergone strict preprocessing.

In summary, existing studies have made notable progress in fine local TLS segmentation, centimeter-scale texture interpretation from oblique photogrammetry, improvements to general-purpose point-cloud networks, and image-domain feature enhancement [32,33]. However, specialized frameworks targeting kilometer-scale UAV oblique-photogrammetry point clouds of sedimentary outcrops remain scarce. Existing works are generally unable to simultaneously address robust segmentation of outcrops with blurred boundaries and irregular geometries, co-adaptation to kilometer-scale scene coverage and low-resolution sparse point clouds, and extreme class imbalance inherent in field surface data. The method proposed in this paper is aimed precisely at filling this gap.

## 3. Methodology

**Figure 1:**
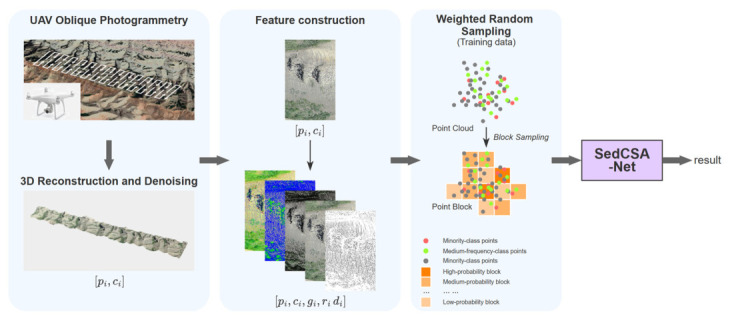
Workflow of the proposed method. The five overlaid images represent the feature vector $\left[p_{i},c_{i},g_{i},r_{i},d_{i}\right]$: position, color, geometry, roughness, and color gradient descriptors for point i.

The proposed sedimentary outcrop point-cloud extraction method consists of three main components (Figure 1): data acquisition and preprocessing, feature construction, and Semantic Segmentation.

First, UAV survey areas are planned in regions where sedimentary outcrops are exposed, and UAV oblique photogrammetry surveys are conducted using a multi-lens oblique camera system. Through multi-view image acquisition with high overlap, continuous RGB imagery covering the study area is obtained. Subsequently, a Structure-from-Motion(SfM) reconstruction workflow is applied to generate point clouds in PLY format containing both spatial coordinates and color information, such that each point is associated with six attributes $\left(x,y,z,R,G,B\right)$. Based on the reconstructed point cloud, artificial structures, outliers, and obvious noise points are removed to obtain a cleaned field-surface point cloud suitable for subsequent deep-learning-based analysis.

Since the original point cloud contains only coordinate and color information and has limited capability for representing sedimentary outcrop boundaries and geological structures, a geological-prior enhancement strategy is introduced to construct multi-source features. This process generates an enhanced input vector that integrates geometric, textural, and color-gradient information. For the i-th point, let its three-dimensional coordinates be $p_{i}=\left(x_{i},y_{i},z_{i}\right)\;$and its RGB color vector be $c_{i}=(R_{i},G_{i},B_{i})$. The enhanced feature representation can then be expressed as

$$x_{i}=[p_{i},c_{i},g_{i},r_{i},d_{i}],$$

where $g_{i}\;$denotes the geometric feature vector composed of attributes such as surface normals and curvature, $r_{i}\;$represents the roughness feature characterizing local surface irregularity and texture variability, and $d_{i}\;$denotes the color-gradient feature describing local color-variation patterns. These features complement one another by capturing the spatial morphology, surface texture, and color differentiation of sedimentary outcrops from multiple perspectives, thereby enhancing the model’s ability to distinguish complex boundaries among outcrops, vegetation, and soil. The detailed computation of these features is described in Section 3.2.

**Figure 2:**
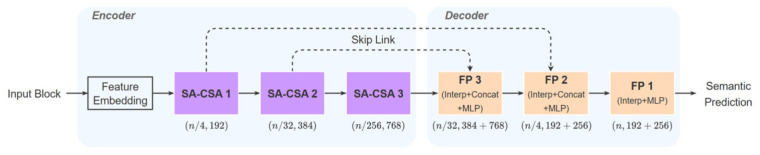
SedCSA-Net architecture.

Based on the enhanced feature representation, the proposed deep-learning architecture, SedCSA-Net (Figure 2), is employed to perform point-wise semantic segmentation of the point cloud. Built on the hierarchical local feature extraction philosophy of PointNet++, the network introduces a cross-scale self-attention(CSA) mechanism within the Multi-Scale Grouping(MSG) module and incorporates a weighted random sampling strategy together with a hybrid loss function during training. These components improve the learning of minority classes and boundary-transition regions, which are common in sedimentary outcrop environments. The network ultimately performs pointwise classification into three semantic categories—outcrop, vegetation, and soil—and produces high-accuracy three-dimensional segmentation results that provide a foundation for subsequent bedding-surface extraction, attitude measurement, and geological interpretation.

### 3.1. UAV Oblique Photogrammetry and 3D Modeling

We use a quadrotor UAV for aerial surveying. For three-dimensional reconstruction, we adopt the standard workflow combining SfM and MVS to recover camera poses from aerial imagery and generate a dense three-dimensional point cloud. The SfM algorithm extracts and matches feature points (e.g., Scale-Invariant Feature Transform(SIFT) features) across multiple images and uses bundle adjustment to simultaneously solve the camera intrinsic and extrinsic parameters and the three-dimensional coordinates of object points; the MVS algorithm then performs dense depth estimation and fusion over all images given the known camera parameters, finally generating a high-density point cloud containing three-dimensional coordinates $\left(x,y,z\right)$ and RGB color information. This study uses DJI Terra v3.7.0 (SZ DJI Technology Co., Ltd., Shenzhen, China) to complete the full reconstruction workflow.

However, large-area field oblique photogrammetry point clouds objectively possess several inherent characteristics: (1) non-uniform point density: under the combined influence of flight height, terrain relief, and image overlap, the point density in valley depressions and on vertical cliff faces is markedly lower than in flat areas; (2) shadow and color distortion: under strong sunlight, shadows produced by terrain occlusion severely distort the color information in some regions; (3) SfM geometric errors: in steep slopes, overhangs, and heavily occluded cliff faces, insufficient image overlap may introduce geometric voids and local deformation during SfM reconstruction. These characteristics are objective concomitants of large-scale field oblique photogrammetry operations and cannot be fully eliminated in practical field deployment. To improve the model’s robustness to real field data, maximize fidelity to actual deployment conditions, and improve overall engineering efficiency, we deliberately do not apply any dedicated treatment to these three data characteristics during preprocessing—i.e., we perform no density homogenization, no shadow removal, and no interpolation to fill geometric voids—so that the training and testing environments remain highly consistent with actual field-deployment conditions, allowing a more objective evaluation of the robustness of SedCSA-Net without preprocessing intervention.

### 3.2. Geological Prior Feature Enhancement

Sedimentary outcrops exhibit distinctive surface characteristics. To enhance the network’s sensitivity to these features, we perform feature enhancement guided by geological domain knowledge.

**Figure 3:**
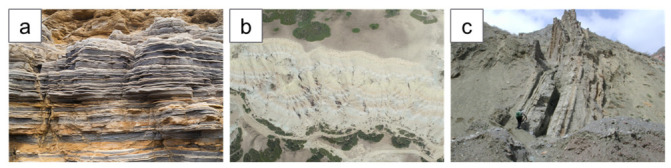
Layered bedding (**a**), color banding (**b**), and differential weathering (**c**) characteristics of sedimentary outcrops.

Sedimentary rocks are layered rocks formed through the weathering, transport, deposition, and diagenesis of materials derived from biological remains, volcanic materials, or other sources. Sedimentary outcrops are portions of sedimentary rocks naturally exposed at the surface, providing direct access to sedimentary environments, lithofacies, paleogeography, and reservoir characteristics. They exhibit three characteristic surface signatures: (a) prominent bedding structures, including laminae, bed sets, and set groups; (b) color banding associated with lithofacies variation; and (c) differential erosional relief produced by selective weathering. Together, these features define the textural and geometric patterns used to identify sedimentary outcrops in three-dimensional space (Figure 3) and provide rich multi-scale visual cues for semantic segmentation.

By integrating the layered structure, color banding, and differential weathering characteristics of sedimentary outcrops, we construct a geological prior-enhanced input vector on top of the raw point cloud:

$$x_{i}=\left[p_{i},c_{i},g_{i},r_{i},d_{i}\right]$$

Here, the geometric feature vector $g_{i}$ consists of surface normals and curvature estimated from the local neighborhood covariance matrix using Principal Component Analysis(PCA); the textural feature $r_{i}$ is a roughness measure defined as the variance of distances from neighborhood points to their best-fit plane; and the color-gradient feature $d_{i}$ captures the spatial partial derivatives of the RGB channels along the x-, y-, and z-directions (9 dimensions in total), representing the spatial distribution of spectral variation. Z-score normalization is applied to $g_{i}$, $r_{i}$, and $d_{i}$ to remove unit differences and ensure a consistent numerical scale during training.

The enhanced input vector is defined as

$$x_{i}=\left[p_{i},c_{i},g_{i},r_{i},d_{i}\right]\;p_{i}=\left(x_{i},y_{i},z_{i}\right)\;c_{i}=\left(R_{i},G_{i},B_{i}\right)\;N_{i}=p_{j}|\left|\left|p_{j}-p_{i}\right|\right|\leq r_{n}\;m_{i}=\left|N_{i}\right|$$

where $p_{i}$ is the 3D coordinate of point i, $c_{i}$ is the RGB color vector, $g_{i}$ is the geometric feature vector, $r_{i}$ is the textural roughness, and $d_{i}$ is the color-gradient feature vector. For a given center point $p_{i}$, its radius neighborhood is defined as $N_{i}$, where $r_{n}$ is the preset neighborhood radius, j indexes the points within the neighborhood, and $m_{i}$ denotes the number of neighborhood points.

First, calculate the geometric Features $g_{i}$, including surface normals and curvature. For each point i, the local covariance matrix within the radius neighborhood $N_{i}$ is computed as

$$C_{i}=\left(1/m_{i}\right)\Sigma_{j\in N_{i}}\left(p_{j}-p{¯}_{i}\right){\left(p_{j}-p{¯}_{i}\right)}^{T}\;p{¯}_{i}=\left(1/m_{i}\right)\Sigma_{j\in N_{i}}p_{j}$$

Eigenvalue decomposition of $C_{i}$ yields the eigenvalues $\lambda_{1}\geq \lambda_{2}\geq \lambda_{3}\geq 0$ and the corresponding unit eigenvectors $v_{1},v_{2},v_{3}$. The eigenvector $v_{3}$, corresponding to the smallest eigenvalue, is used as the PCA estimate of the local surface normal:

$$n_{i}=v_{3}\;\kappa_{i}=\lambda_{3}/\left(\lambda_{1}+\lambda_{2}+\lambda_{3}\right)\;g_{i}=\left[n_{i};\kappa_{i}\right]\in R^{4}$$

Secondly, extract the texture roughness feature textural roughness $r_{i}$ witch is defined as the mean squared distance from local neighborhood points to the fitted plane:

$$r_{i}=\left(1/m_{i}\right)\Sigma_{j\in N_{i}}{\left(n_{i}^{T}\left(p_{j}-p{¯}_{i}\right)\right)}^{2}$$

This measure shows strong regularity on rough exposed outcrop surfaces formed by differential weathering, particularly within individual sedimentary layers where roughness values tend to be homogeneous. It therefore effectively characterizes surface textural irregularity and helps delineate continuous sedimentary layers.

Thirdly, to capture the spatial distribution of color banding in sedimentary outcrops, local color gradients are estimated for each RGB channel along the x-, y-, and z-directions. For the channel c∈{R,G,B} and direction δ∈{x,y,z}, the partial derivative is estimated using a distance-weighted difference within the radius neighborhood:

$${\left.\frac{\partial c}{\partial \delta }\right|}_{p_{i}}\approx \frac{\sum_{j\in N_{i}}w_{ij}\left(c_{j}-c_{i}\right)\left(\delta_{j}-\delta_{i}\right)}{\sum_{j\in N_{i}}w_{ij}{\left(\delta_{j}-\delta_{i}\right)}^{2}+\varepsilon },\;w_{ij}=exp\left(-{\left|\left|p_{j}-p_{i}\right|\right|}^{2}/\left(2\sigma^{2}\right)\right)$$

where $w_{ij}$ are Gaussian weights, σ is the mean neighborhood distance, and ϵ is a small constant for numerical stability. The color-gradient feature is formed by concatenating the gradients of the three channels along the three spatial directions: $d_{i}\in \;R^{9}$.

Finally, to eliminate unit differences among features, the mean $\mu_{f}$ and standard deviation $\sigma_{f}$ are computed on the training sets for $g_{i}$, $r_{i}$, and $d_{i}$, respectively, followed by Z-score normalization:

$$\hat{f}=\left(f-\mu_{f}\right)/\left(\sigma_{f}+\varepsilon \right),\varepsilon =10^{-6}$$

### 3.3. Weighted Random Sampling Strategy

Sedimentary outcrops in the field are commonly covered by extensive vegetation and soil [15], resulting in a severe class imbalance between outcrops, vegetation, and soil (approximately 1:4:9 in our dataset). Conventional uniform sampling may cause minority-class samples to be overwhelmed during training, leading the model to misclassify outcrop points as soil or vegetation near class boundaries.

To alleviate this issue, we introduce a weighted random sampling strategy based on the proportion of rare-class points during training. The entire training point cloud is partitioned into non-overlapping spatial blocks $B_{i}$ of fixed side length L. For each block $B_{i}$, the proportion of rare-class (outcrop) points is computed as

$$f_{\mathrm{minority},i}=\frac{\#\{\;p\in B_{i}:y(p)=c_{\mathrm{rare}}\;\}}{\#B_{i}}$$

The sampling weight for block $B_{i}$ in each epoch is then defined as

$$W_{i}={\left(f_{minority,i}+\varepsilon \right)}^{\tau }$$

where ϵ=0.001 ensures that blocks containing no rare-class points still retain a non-zero minimum sampling probability, and *τ*=1.0 controls the strength of the sampling bias. Sampling probability is proportional to $W_{i}$, and a fixed number of blocks is sampled without replacement from all blocks in each epoch according to these weights.

This strategy increases the exposure frequency of rare-class samples at a controllable cost, enabling the network to learn more discriminative features for the outcrop class while maintaining reasonable coverage of the other classes.

### 3.4. Multi-Scale Feature Encoder with Cross-Scale Attention Enhancement

To address the varying exposure conditions and wide range of feature scales characteristic of sedimentary outcrops, SedCSA-Net adopts Multi-Scale Grouping (MSG) in each Set Abstraction(SA) layer to extract local features at multiple radii $R=[r_{1},r_{2},\ldots ,r_{S}]$ centered on the sampled point $p_{c}$, yielding S scale branches. To facilitate cross-scale interaction, the features from each branch are mapped to a unified channel dimension. The s-th scale branch at the l-th SA layer is denoted as $F_{l}^{s}\in R^{M\times C}$, where M is the number of sampled center points and C is the number of channels. Standard MSG concatenates $F_{l}^{s}$ along the channel dimension without distinguishing the geological importance of different scales.

**Figure 4:**
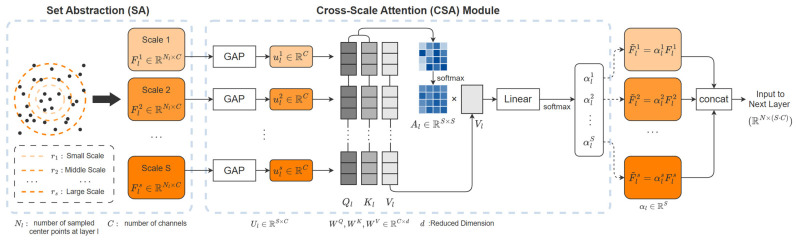
Architecture of the SA–CSA module.

To assign higher weights to scales more relevant to the current task, a cross-scale self-attention(CSA) module is embedded above MSG, forming the SA–CSA module shown in Figure 4.

For the S scale branches $F_{l}^{s}$ at the l-th SA layer, CSA first extracts a state descriptor for each branch via global average pooling:

$$u_{l}^{s}=\left(1/N\right)\Sigma_{n}F_{l}^{s}\left[n,:\right]\in R^{C}\;U_{l}=\left[u_{l}^{1};u_{l}^{2};\ldots ;u_{l}^{S}\right]\in R^{S\times C}$$

Query, key, and value are then constructed by linear projection as follows:

$$Q_{l}=U_{l}W_{Q}\;K_{l}=U_{l}W_{K}\;V_{l}=U_{l}W_{V}$$

where $W_{Q}$, $W_{K}$, and $W_{V}\in R^{C\times d}$, and d is the scaling dimension. The inter-scale attention matrix is

$$A_{l}=softmax\left(Q_{l}K_{l}^{T}/\sqrt{d}\right)\in R^{S\times S}$$

A weighted sum over $V_{l}$ yields the recalibrated scale states as follows:

$$U_{l}^{\prime }=A_{l}V_{l}\in R^{S\times d}$$

Scalar weights for each scale are obtained by applying a linear projection and Softmax to $U_{l}^{\prime }$:

$$\alpha_{l}=softmax\left(U_{l}^{\prime }w_{O}+b_{O}\right)\in R^{S}$$

where $w_{O}\in R^{d}\;$and $b_{O}\in R^{S}$. The final reweighted scale branches are

F^ls=αlsFls,s=1,…,S

where $\alpha_{l}^{s}$ is the scalar attention weight for scale s. The reweighted multi-scale features are concatenated along the channel dimension and passed to the next layer.

This “grouped extraction–attention fusion” design preserves the computational efficiency of PointNet++ while enabling the model to adaptively adjust its effective receptive field for different field features: for large, continuous outcrops, CSA tends to amplify larger-scale branches to capture layered band structures; for fragmented, scattered small outcrops, it tends to strengthen smaller-scale branches to preserve detailed edge geometry.

### 3.5. Hybrid Loss Function

Class imbalance and boundary accuracy jointly determine the success of sedimentary outcrop segmentation. Although weighted sampling addresses imbalance from the data side, the loss function must further guide the model to focus on hard-to-classify samples and fine boundaries. We combine Focal–Tversky loss [11] and Lovász–Softmax loss [12] using the following weighted sum:

$$L=\Omega_{1}L_{F}T+\Omega_{2}L_{L}ovász$$

where $\lambda_{1}\;$and $\lambda_{2}$ are non-negative scalar hyperparameters ($\Omega_{1}=\Omega_{2}=0.5$ in this work).

(1) Focal–Tversky Loss

For class c, the Tversky index is defined as

$$T_{c}=TP_{c}/\left(TP_{c}+\alpha_{T}FN_{c}+\beta FP_{c}\right)$$

where $TP_{c}$, $FN_{c}$, and $FP_{c}$ are the true positive, false negative, and false positive point counts for class c, respectively; $\alpha_{T}$ and β control the relative penalties for false negatives and false positives. The Focal–Tversky loss is

$$L_{F}T=\left(1/\left|C\right|\right)\Sigma_{c\in C}{\left(1-T_{c}\right)}^{\gamma }$$

where γ=4/3 is the focal parameter and ∣C∣ is the number of classes. This term increases the focus on hard-to-classify samples with small $T_{c}$. Class averaging also avoids scale fluctuations caused by changes in the number of classes.

(2) Lovász–Softmax Loss

Berman et al. [10] showed that the Lovász extension provides a tight convex surrogate for the discrete IoU in continuous probability space. Let $p_{i,c}$ denote the per-point Softmax probability for class c. For each point i, the error for class c is defined as

$$e_{i,c}=1\left[y_{i}=c\right]\left(1-p_{i,c}\right)+1\left[y_{i}\neq c\right]p_{i,c}$$

After sorting $e_{i,c}$ in descending order, the Lovász–Softmax loss for class c is

$$L_{Lovász,c}=\Sigma_{i}e_{\left(i\right)},c\cdot \Delta J_{c}\left(i\right)$$

where $\Delta J_{c}\left(i\right)\;$is the marginal term of the Lovász extension corresponding to the Jaccard loss $\left(1-IoU\right)$ for class c. The final loss is

$$L_{L}ovász=\left(1/\left|C\right|\right)\Sigma_{c\in C}L_{Lovász,c}$$

This loss directly optimizes the IoU evaluation metric and is particularly effective for fine-grained recognition of outcrop boundaries and fragments.

The hybrid loss emphasizes hard samples and rare classes at the sample level through Focal–Tversky loss, while aligning with the IoU optimization objective at the metric level through Lovász–Softmax loss. Together with weighted sampling, it forms a two-pronged mechanism for handling class imbalance from both the sampling and loss perspectives.

## 4. Dataset

### 4.1. Data Acquisition and Preprocessing

The study data were collected in the Louzhuangzi area of the southern Junggar Basin. Tectonically, Louzhuangzi is situated in the northern Tianshan piedmont depression [16], along the eastern segment of the southern Junggar Basin margin, bounded by the Louzhuangzi syncline to the south and the Kalaza syncline to the north, with stratum strikes oriented north–northeast [17]. The area encompasses Jurassic, Cretaceous, Paleogene, and Neogene strata. This study focuses on the Cretaceous Qingshuihe Formation outcrops, which are predominantly composed of medium-to-coarse-grained sandstone [18] and have been naturally sculpted by gully erosion under southern uplift.

The outcrops of the Qingshuihe Formation exhibit diverse morphologies [19]. In terms of exposure, they range from extensively bare outcrops to heavily vegetation- or soil-covered surfaces, as well as small, fragmental blocks. Regarding exposure position, outcrops appear as vertically protruding ridges on hillsides or hilltops and as incised sections formed by gully erosion. Outcrop scales vary substantially, with exposed lengths ranging from tens of centimeters to over a hundred meters. This morphological and scale diversity makes the Louzhuangzi Qingshuihe Formation an ideal site for developing and evaluating outcrop point-cloud segmentation methods.

**Figure 5:**
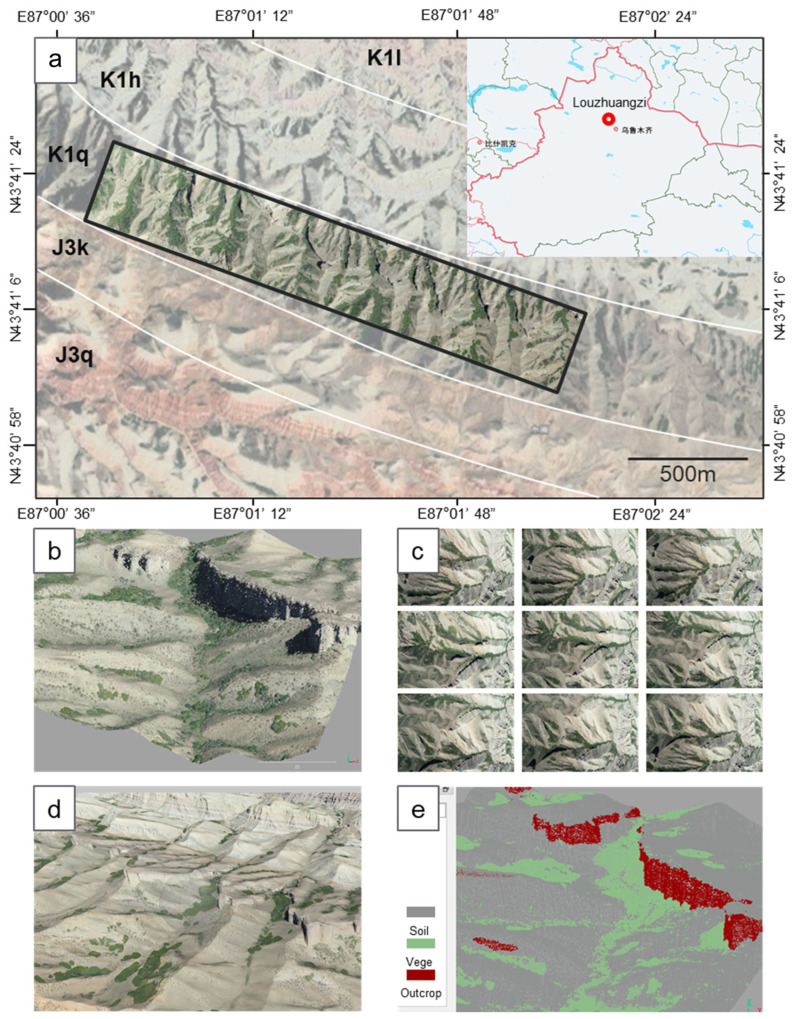
Workflow for dataset construction. (**a**) Dataset acquisition area; (**b**) point cloud; (**c**) original oblique-photogrammetry image; (**d**) raster model; (**e**) category annotation.

A rectangular study area was delineated along the stratum strike, measuring 2073.9 m in length and 333.4 m in width, with a maximum elevation difference of 166.9 m, and covering a projected area of 691,488 m^2^ (Figure 5a). UAV oblique photogrammetry was conducted with a DJI Phantom 4 RTK (SZ DJI Technology Co., Ltd., Shenzhen, China) equipped with an FC6310R survey camera (20 MP, 1-inch CMOS, 24 mm equivalent focal length). Flights were conducted at 200 m altitude with forward and side overlaps of 80% and 70%, respectively. Real-Time Kinematic(RTK) positioning was adopted, with coordinates referenced to CGCS2000/3-Degree Gauss–Krüger CM 87E. A total of 159 images were captured and processed using DJI Terra for 3D reconstruction, generating point-cloud data. Edge areas with irregular boundaries and insufficient overlap were trimmed to produce a geometrically stable and uniformly distributed point cloud.

Data preprocessing in this paper mainly performs denoising of the point cloud: the initial reconstruction included man-made structures (e.g., utility poles, buildings, livestock enclosures) and floating or isolated noise. These were manually removed in CloudCompare v2.12 (Daniel Girardeau-Montaut, open-source; available at https://www.cloudcompare.org, 20260601 (accessed on 11 June 2026)), followed by automated cleaning using statistical outlier removal and small-cluster elimination to minimize non-geological information that could interfere with model training.

**Figure 6:**
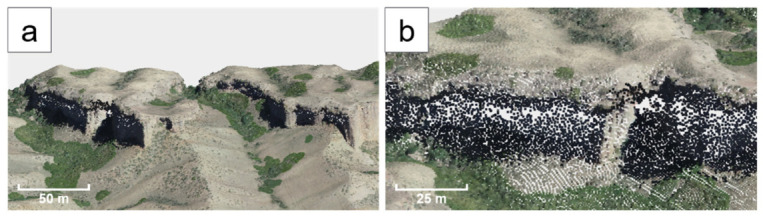
Shadows (**a**) and voids/non-uniform density (**b**) in the raw point cloud.

To improve the model’s robustness to real field data, maximize fidelity to actual deployment conditions, and improve overall engineering efficiency, we deliberately do not apply dedicated treatment to the three data characteristics inherent in oblique photogrammetry point clouds during preprocessing: we do not perform density homogenization (i.e., do not unify point spacing across regions), we retain shadow-induced color-distortion regions, and we do not interpolate to fill the geometric voids formed during SfM reconstruction (Figure 6). These three phenomena are unavoidable objective concomitants of large-area field oblique photogrammetry operations; although dedicated treatment can improve local data quality to some extent, it also introduces additional parameter-tuning burdens and may mask the practical challenges that the model faces in real scenes. Retaining these raw data characteristics keeps the training and testing environments highly consistent with actual field-deployment conditions, allowing a more objective evaluation of the robustness of SedCSA-Net without preprocessing intervention and verifying its practical usability on kilometer-scale field point clouds.

### 4.2. Dataset Annotation and Partitioning

The three-dimensional morphology of geological outcrops is complex and diverse, and point clouds at the boundaries of outcrops, soil, and vegetation are prone to misjudgement if annotated using point-cloud RGB color information alone. Therefore, this study adopts multi-source cross-validation for annotation. To improve annotation accuracy, a multi-source cross-validation strategy was adopted, combining point-cloud geometric (XYZ) and color (RGB) information with original UAV photographs and oblique-photogrammetry raster models (Figure 5). For the outcrop–soil boundary, the discrimination relies mainly on abrupt changes in local point-cloud curvature and surface roughness, combined with rock–soil boundaries in the original UAV imagery; the outcrop–vegetation boundary focuses on three-dimensional morphological differences and color contrast, using the texture of the oblique photogrammetry raster model to discriminate vegetation-attachment edges; the vegetation–soil boundary is determined comprehensively from point-cloud elevation, surface smoothness, and vegetation-patch outlines in the original imagery. All boundary-ambiguous points are verified by tracing back to multi-source imagery and then spot-checked and corrected by geological experts. This approach compensates for locally missing textures and enhances the identification of features such as exposed stratum boundaries.

Initial annotations were reviewed through sampling inspections and boundary corrections by experienced geologists. The final class distribution among outcrops, vegetation, and soil was approximately 1:4:9. The outcrop class, as the minority class, presents a particular challenge for recognition, making it a suitable benchmark for evaluating the robustness of the segmentation network.

**Figure 7:**
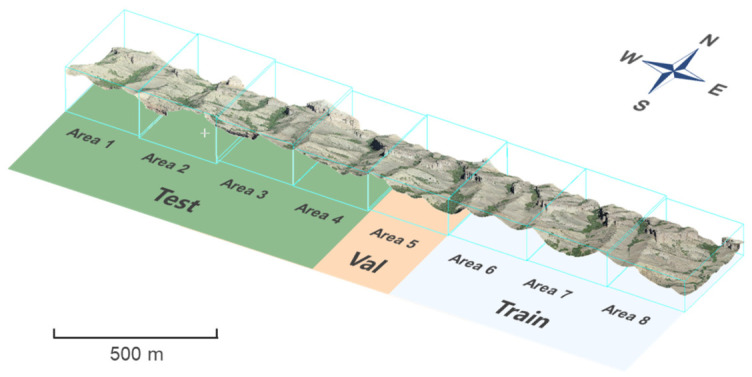
Dataset partitioning.

Compared with previous sedimentary or lithological studies, the dataset partitioning strategy was optimized for large-scale field surveys. Prior approaches often subdivided a single outcrop into small, regular grid blocks, each containing a few hundred points, with training and testing conducted on these blocks. While suitable for fine-grained recognition of small-scale outcrops, this method does not adequately capture the spatial distribution of large-scale outcrops. In practice, sedimentary outcrops can extend over vast areas, whereas field access is often limited. To address this, the entire outcrop was divided into eight independent spatial blocks along the principal strike (Regions 1–8; Figure 7) [20]. A spatially progressive split was applied for training (Regions 6–8), validation (Region 5), and testing (Regions 1–4), with a train/validation/test ratio of 3:1:4. This extrapolation-style partitioning evaluates model generalization under variations in geological dip direction and angle.

## 5. Experiments and Results

This section presents the experimental setup, evaluation metrics, ablation study, comparative experiments, and geological application analysis of the segmentation results, with the aim of verifying the effectiveness and robustness of SedCSA-Net in complex sedimentary outcrop environments.

### 5.1. Experimental Setup and Evaluation Metrics

The experimental hardware platform comprised an AMD R7-5700X CPU, 64 GB of RAM, and an NVIDIA RTX 2080 Ti GPU with 11 GB of VRAM. The software environment was 64-bit Windows 10, and PyTorch 2.4.1 and CUDA 11.8 were used for model training and inference. During training, the Adam optimizer was adopted with an initial learning rate of 0.001 and a weight decay of 1 × 10^−4^. A stepwise learning-rate decay strategy was applied, reducing the learning rate by a factor of 0.8 every 20 epochs. The model was trained for 200 epochs with a batch size of 16.

Given the large spatial extent of outcrops in the field, the original point cloud was first partitioned into fixed-size spatial blocks. Based on the exposure scale of the Qingshuihe Formation outcrops in Louzhuangzi, each block was assigned a side length of 100 m, and 4096 points were randomly sampled from each block as network input to preserve local geometric integrity while limiting GPU memory consumption. During training, a weighted random sampler based on the proportion of rare-class points assigned different sampling weights to individual blocks, thereby substantially increasing the probability of selecting blocks containing more outcrop points. During validation and testing, uniform random sampling was used to ensure objective and comparable evaluation results.

To further alleviate class imbalance, a class-weighted loss function was used during training, where α = 0.7 and β = 0.3, imposing a larger penalty on misclassification of rare classes. This strategy effectively improved segmentation performance for rare classes while maintaining Overall Accuracy.

Three evaluation metrics were used to comprehensively assess model performance in complex sedimentary outcrop scenarios: mean Intersection over Union (mIoU), per-class Intersection over Union (IoU), and Overall Accuracy (OA).

Mean Intersection over Union (mIoU) is a widely used comprehensive metric in semantic segmentation, defined as follows:

$$mIoU=\frac{1}{C}\sum_{c=1}^{C}IoU_{c}\;IoU_{c}=\frac{TP_{c}}{TP_{c}+FP_{c}+FN_{c}}$$

where $TP_{c}$, $FP_{c}$, and $FN_{c}$ denote the true positive, false positive, and false negative point counts for class c, respectively, and C is the total number of classes. From a geoscience perspective, a higher mIoU indicates a stronger overall ability of the model to distinguish outcrops, vegetation, and soil in the field.

Per-class IoU is of particular importance because the primary objective of this study is sedimentary outcrop extraction. A higher IoU indicates better geometric fidelity in outcrop recognition.

Overall Accuracy (OA) is the proportion of all correctly classified points, reflecting the overall prediction accuracy of the model and providing a macroscopic measure of correctness across the entire dataset.

In addition, the segmentation results were evaluated from a geoscience application perspective in terms of boundary fidelity, continuity of outcrop preservation, and usability for downstream geometric measurements.

### 5.2. Ablation Study and Baseline Comparison

To validate the effectiveness of each improvement module in SedCSA-Net, five progressive ablation experiments were conducted on Regions 1–4. Each experiment was repeated five times, and the results were averaged. The results are shown in Table 1.

As shown in Table 1, the sequential incorporation of geological prior feature enhancement, weighted random sampling, the CSA module, and the hybrid loss function consistently improved performance, indicating strong synergistic effects among these components. The baseline achieved an mIoU of 82.91%, an OA of 89.50%, and an outcrop IoU of 75.69%. After introducing geological prior features, these metrics improved to 84.47%, 90.82%, and 78.63%, respectively, demonstrating that surface normals, curvature, roughness, and color-gradient features effectively enhanced the model’s ability to distinguish outcrops from other classes. Incorporating weighted random sampling increased the outcrop IoU from 78.63% to 82.55%, confirming that increasing the sampling weight of rare-class blocks helps alleviate the class imbalance. The addition of the CSA module produced the most substantial improvement: mIoU reached 88.21%, OA reached 95.02%, and outcrop IoU reached 84.94%, highlighting the critical role of its adaptive cross-scale feature fusion in complex sedimentary outcrop segmentation. Finally, with the additional constraint of the hybrid loss function, SedCSA-Net achieved the best overall performance.

In summary, the four modules do not merely provide additive gains; rather, they cooperate to improve model performance from four complementary perspectives: feature representation, sample distribution, multi-scale fusion, and boundary optimization. The largest gains were observed in correct outcrop-class segmentation. Moreover, the performance gain on introducing each module is monotonically increasing, but it is of different magnitudes—weighted random sampling gives the most prominent marginal improvement in outcrop IoU, while the CSA module contributes most to mIoU and OA—indicating that the modules’ contributions are distinguishable and mutually non-masking, with substantive independent effects.

### 5.3. Comparative Experiment Analysis

**Figure 8:**
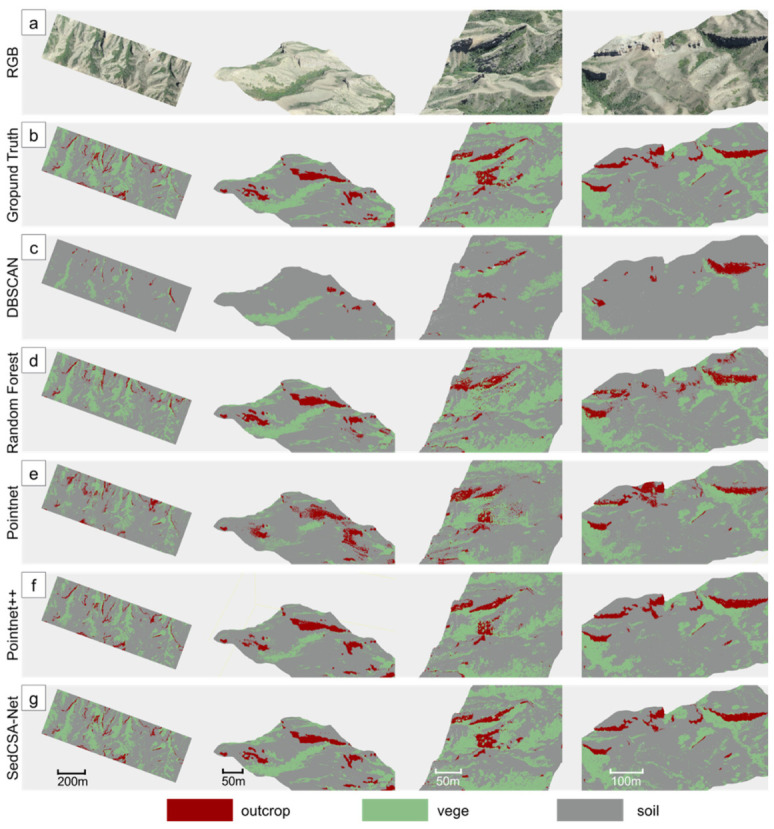
Comparison of segmentation results produced by different methods. (**a**) Original point cloud; (**b**) ground-truth annotation; (**c**) DBSCAN; (**d**) Random Forest; (**e**) PointNet; (**f**) PointNet++; (**g**) Proposed SedCSA-Net.

To further validate the effectiveness of SedCSA-Net, Density-Based Spatial Clustering of Applications with Noise(DBSCAN), Random Forest, PointNet, and PointNet++ were selected as baseline methods. All models were trained, validated, and tested using identical data splits, and the averaged results from test Regions 1–4 are reported in Table 2 and Figure 8.

Overall, the methods exhibited markedly different performance in complex sedimentary outcrop scenarios. DBSCAN achieved only 37.37% mIoU and 15.73% outcrop IoU, indicating that density-based clustering is poorly suited to the spatially interleaved distribution of outcrops, soil, and vegetation in field settings. Random Forest achieved an OA of 88.21% but only 63.77% mIoU and 19.48% outcrop IoU. As shown in Figure 8d, this method identified vegetation relatively well (IoU of 87.75%) but showed substantial confusion between outcrops and soil, together with pronounced salt-and-pepper artifacts, suggesting that color-based methods lack 3D structural modeling capability and cannot capture outcrop continuity. PointNet achieved 38.86% mIoU, 25.81% outcrop IoU, and 66.11% OA. Its segmentation results were generally blurred (Figure 8e), with poor performance at outcrop boundaries and on small, exposed areas because it relies primarily on global feature representations without sufficient local geometric modeling. PointNet++, which introduces hierarchical local feature learning, achieved substantially better performance, with an mIoU of 82.91%, an outcrop IoU of 75.69%, and an OA of 89.50%. As shown in Figure 8f, this method recovered the overall spatial distribution of outcrops but still exhibited under-segmentation and misclassification in small-scale outcrops and transition zones between outcrops, soil, and vegetation, with boundary delineation that remained insufficiently precise.

SedCSA-Net achieved the best performance across all metrics, with an mIoU of 89.51%, an outcrop IoU of 86.90%, vegetation and soil IoUs of 89.69% and 91.95%, respectively, and an OA of 96.08%. As shown in Figure 8g, SedCSA-Net produced segmentation results that most closely matched the ground truth. At the macroscopic level, the model accurately recovered the continuous band-like distribution of outcrops along the stratum strike. At the local level, it effectively identified small, fragmented outcrops while maintaining clear boundaries.

### 5.4. Robustness Analysis to the Natural Variability of UAV-SfM Data

**Figure 9:**
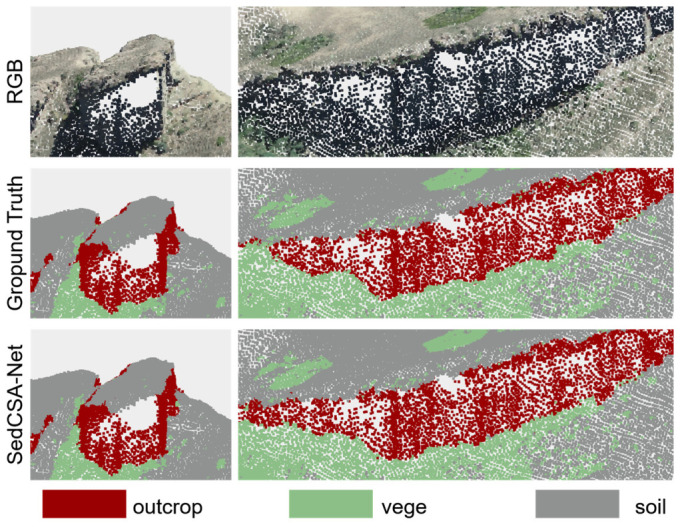
Examples of segmentation robustness in shadowed and geometric-void regions.

Figure 9 shows two cliff outcrops where three-dimensional reconstruction produced voids, non-uniform density, and shadows. As can be seen, the large voids and unevenly distributed points in the point cloud do not noticeably interfere with the segmentation results of SedCSA-Net; in these regions, the model’s segmentation remains highly consistent with the ground truth. At the shadowed cliff base, a small number of outcrop–vegetation points are misclassified, but the overall boundary is complete, with no regional collapse. This is attributable, on the one hand, to the color-gradient features being insensitive to absolute illumination intensity and preserving the spatial relative-change pattern; and, on the other hand, to the cross-scale attention mechanism enabling the model to borrow texture information from the undistorted regions at shadow edges, thereby maintaining robust segmentation within shadowed areas. These results show that even without any preprocessing of the voids and shadows in UAV-SfM data, SedCSA-Net retains good robustness to natural variability.

### 5.5. Geological Measurement Validation Based on Segmentation Results

To further validate the usability of SedCSA-Net segmentation results for geological interpretation, we extracted the boundaries of segmented outcrop regions and identified continuous sedimentary stratum interfaces. Least-squares plane fitting was then applied separately to the interface point sets extracted from the manually annotated dataset and from the SedCSA-Net segmentation results, and the corresponding stratigraphic attitudes were computed. The results are shown in Figure 8 and Table 3.

**Figure 10:**
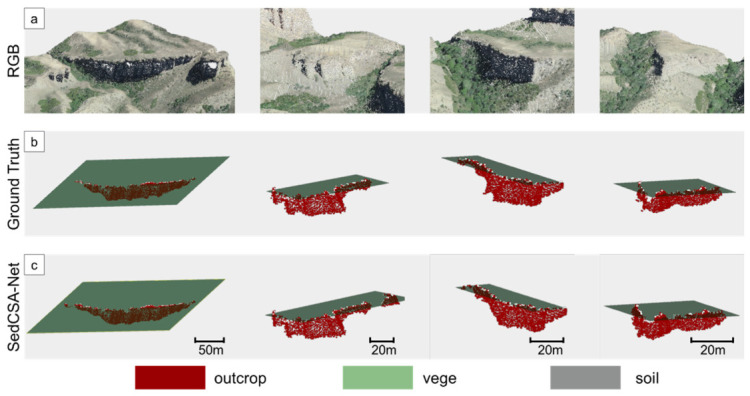
Comparison of fitted sedimentary layer boundaries. (**a**) Original point cloud; (**b**) manually annotated reference fitting surface; (**c**) fitting surface derived from SedCSA-Net segmentation results.

As shown in Figure 10, the SedCSA-Net segmentation results and the manually annotated reference agree closely in terms of stratum interface position and overall spatial distribution, preserving the geometric continuity of sedimentary layer boundaries. Table 3 shows that the dip-direction and dip-angle errors between the attitudes fitted from SedCSA-Net segmentation results and those derived from manual annotations are all within 3°, confirming the reliability of the method for quantitative geometric analysis of sedimentary stratum interfaces. These results demonstrate that SedCSA-Net produces geologically interpretable and geometrically consistent outcrop recognition and can further support measurements of stratigraphic attitudes, bedding tracing, and sedimentary architecture analysis in the southern Junggar Basin.

## 6. Discussion

### 6.1. Implications of UAV Oblique Photogrammetry for Sedimentary Outcrop Interpretation

UAV oblique photogrammetry provides an efficient means of acquiring kilometer-scale sedimentary outcrop data and forms the basis of the workflow proposed in this study. Compared with TLS, it enables rapid coverage of extensive outcrop corridors while simultaneously preserving geometric structure and color information. These characteristics make it particularly suitable for large-scale sedimentological investigations, including stratigraphic tracing, outcrop mapping, and sedimentary architecture analysis.

However, UAV-SfM point clouds also exhibit substantial natural variability caused by illumination conditions, viewing geometry, and reconstruction uncertainty. In field environments, these effects commonly appear as shadowed regions, non-uniform point density, reconstruction voids, and local geometric artifacts, all of which may reduce segmentation accuracy. The robustness analysis presented in Section 5.4 shows that SedCSA-Net remains effective under such conditions, maintaining consistent segmentation results even in areas affected by shadows and geometric incompleteness. This robustness is attributed to the combined effects of geological-prior feature enhancement and cross-scale feature aggregation, which enable the model to exploit both local and contextual information.

These results suggest that the proposed framework is well suited to the characteristics of real UAV-SfM geological datasets and can provide reliable semantic information for subsequent geological applications, including bedding-surface extraction, attitude measurement, and sedimentary architecture characterization.

### 6.2. Sources of Synergistic Contribution from Framework Modules

This study treats sedimentary outcrop point-cloud interpretation as an integrated pipeline encompassing acquisition, feature engineering, sampling, encoding, loss design, and downstream application, with each stage explicitly adapted to the physical and statistical characteristics of sedimentary outcrops. The ablation study (Table 1) shows that the four improved modules contribute IoU gains of 2.94% from geological prior features, 3.92% from weighted random sampling, 2.39% from the CSA module, and 1.96% from the hybrid loss, resulting in a total improvement of 11.21% (from 75.69% to 86.90%). This progressive improvement reflects more than simple metric accumulation; its mechanism can be explained from three perspectives.

First, prior geological feature enhancement and weighted random sampling substantially expand the discriminative capacity for the outcrop class at the input stage. PCA-derived surface normals and curvature capture local geometric variations at bedding surfaces and differentially weathered interfaces; roughness characterizes textural irregularity on weathered exposed surfaces; and color gradients encode the spatial distribution of color banding in sedimentary rocks. Together, these features provide the model with 14 additional dimensions of discriminative information beyond raw RGB + XYZ.

Second, the CSA module provides adaptive receptive fields for multi-scale features at the intermediate stage. As shown in Figure 7, PointNet++ performs well on large, continuous outcrops but still exhibits substantial under-segmentation in small, fragmented outcrops and at outcrop–soil transition zones. CSA applies soft attention to reweight the global statistics of each scale branch, enabling the model to automatically select the most appropriate scale combination for the dominant features in the current block, thereby improving both the completeness of large-scale spatial distributions and the delineation of small-scale boundaries.

Third, weighted random sampling and the hybrid loss function form a dual mechanism for mitigating class imbalance at the output stage: the former increases the frequency with which the model encounters rare-class samples by adjusting the data distribution, while the latter directly optimizes IoU and imposes additional penalties for missed outcrop samples. Their synergistic effect explains why the hybrid loss still delivers a 1.96% improvement in outcrop IoU even after the sampling strategy has been introduced.

### 6.3. Comparison with Existing Sedimentary Outcrop Point-Cloud Interpretation Work

Compared with existing TLS-based outcrop interpretation studies [3,20,21,22,23,24,25,26,31,32,33], the most fundamental distinction of our framework is that it targets kilometer-scale UAV oblique-photogrammetry point clouds, with limited resolution, large-scale scenes, and severe class imbalance as the core design constraints. Liu et al. [22] (MC-H-Geo) and Duan et al. [26] (FPCS) achieved high-precision fine-grained lithological classification on TLS point clouds using multi-scale contextual features and compressive sampling. Gan et al. [21] (SCCC) achieved an OA of 94.64% on the Qingshuihe Formation by exploiting stratigraphic continuity constraints. These studies focus on fine-grained lithological subdivision at the decameter scale and are complementary to our objective of kilometer-scale semantic segmentation of outcrop–vegetation–soil classes followed by attitude measurement. In short, TLS-based methods emphasize small-scale, high-precision interpretation, whereas our framework emphasizes large-scale, high-throughput processing.

Compared with oblique photogrammetry-based studies [4,27,28,29,30,31,34], existing methods largely rely on centimeter-level texture inputs or segmentation of 2D images before projecting the results back to 3D. Roemers-Oliveira et al. [27] and Bemis et al. [34] achieved high lithofacies classification accuracy through CNN-based image segmentation and 3D back-projection, but their pipelines remain discontinuous between 2D and 3D and cannot fully exploit 3D geometric information. Chen et al. [4,27] directly segment strata in voxelized point clouds using 3D CNNs, but their work primarily targets centimeter-scale sections with relatively balanced class distributions. In contrast, under a three-class imbalanced scenario with decimeter-level resolution and a point ratio of 1:4:9, the present study improves outcrop IoU to 86.90% and automatically measures attitude with errors within 3°, demonstrating strong practical applicability.

### 6.4. Geoscience Application Value and Limitations

From a geoscience application perspective, this framework directly supports three downstream tasks: (1) large-area sedimentary stratum interface tracing, where accurate identification of the outcrop–cover boundary improves the corresponding metric by 11.21% over PointNet++; (2) attitude measurement, where least-squares plane fitting of segmented point sets yields dip direction and dip angle errors within 3°, matching the precision of field compass measurements and supporting stratigraphic attitude mapping and sedimentary architecture analysis; and (3) reservoir heterogeneity characterization, where the exposure degree and geometric morphology of different outcrops are preserved and can serve as geometric input for reservoir architecture analog studies.

However, the proposed framework still has several limitations. First, the experimental data were collected from a single area with a single lithological assemblage, dominated by medium-to-coarse sandstone; the transferability of the framework to carbonate rocks, volcaniclastics, and multilithological combinations remains to be verified. Different lithologies may exhibit distinct textural, color, and geometric characteristics to which the current model may be sensitive. Second, multispectral or hyperspectral data were not incorporated; color gradients were constructed only in the visible spectrum [35,36], making it difficult to distinguish geological units with similar lithology but different mineralogical compositions [37,38]. For interbedded limestone–sandstone or volcaniclastic–shale sequences, RGB features alone may not provide a reliable basis for classification [39]. Third, the framework currently produces only three semantic labels—outcrop, vegetation, and soil—without fine-grained recognition of lithofacies subclasses, structural elements (joints, faults, and fractures), or sedimentary units [40], which limits deeper geological interpretation of tectonic and depositional environments [41]. Fourth, the comparative experiments in this study were primarily conducted against traditional machine-learning methods and classical deep-learning models for point-cloud analysis, while more recent advanced point-cloud semantic segmentation networks were not systematically evaluated. Owing to the lack of a unified public benchmark dataset for geological outcrop point clouds, the applicability of different models in this domain remains to be further validated. Future work will involve more extensive benchmarking experiments to comprehensively assess the generalization capability of the proposed framework.

## 7. Conclusions and Future Work

This study presents an end-to-end intelligent interpretation framework for kilometer-scale sedimentary outcrop point-cloud interpretation from UAV oblique photogrammetry data. The framework integrates UAV oblique photogrammetric data acquisition, geological prior feature enhancement, rare-class-aware sampling, cross-scale self-attention-based multi-scale encoding, hybrid-loss optimization, and automatic attitude measurement from segmentation results into a unified pipeline. Its core module, sedimentary cross-scale self-attention network (**SedCSA-Net**), incorporates four collaborative improvements over PointNet++, systematically adapting the model to sedimentary outcrop segmentation from four perspectives: feature representation, sample distribution, multi-scale fusion, and boundary optimization.

Using the Cretaceous Qingshuihe Formation in the Louzhuangzi area of the southern Junggar Basin as a case study, the proposed framework achieves an mIoU of 89.51% and an OA of 96.08% on the test set, with an outcrop-class IoU of 86.90%, significantly outperforming DBSCAN, Random Forest, PointNet, and PointNet++. The dip direction and dip angle of the least-squares-fitted stratum interfaces derived from the segmentation results both deviate by less than 3° from the manually annotated reference values. Overall, the proposed framework demonstrates strong practical utility for intelligent segmentation, geometric reconstruction, and attitude extraction from large-scale sedimentary outcrop point clouds, and it can support downstream geological tasks such as stratigraphic correlation, attitude measurement, and sedimentary architecture analysis.

Future work may focus on the following directions:

Cross-region generalization and large-scale interpretation: It would be beneficial to extend the dataset to representative sedimentary outcrop scenarios across the Junggar, Tarim, and Sichuan basins, and establish a publicly available benchmark covering diverse lithological assemblages to systematically evaluate model transferability and robustness. Building on this foundation, integrate segmentation results with geological semantic alignment to enable multi-outcrop joint analysis, sedimentary-unit continuity assessment, and regional-scale 3D geological modeling with reduced manual intervention.

Multi-source data fusion: In addition to RGB point clouds, we want to incorporate multispectral/hyperspectral imagery, TLS or airborne LiDAR intensity data, and digital elevation models (DEMs) to construct a unified multi-source, multi-scale, multimodal feature representation, thereby improving lithological discrimination and segmentation accuracy in complex depositional environments.

Fine-grained lithofacies and structural recognition: Building on the existing three-class framework, it would be possible to introduce lithofacies subclass labels (e.g., fine sandstone, coarse sandstone, conglomerate, and mudstone), structural elements (faults, joints, fractures), and sedimentary-unit labels. Hierarchical expert classification combined with stratigraphic continuity constraints could then be used to achieve higher-level geological semantic understanding.

Advanced model benchmarking and evaluation: Building upon the expanded geological benchmark datasets, future work will systematically evaluate SedCSA-Net against more recent point-cloud semantic segmentation architectures. Such comparisons will help clarify the advantages and limitations of different approaches for kilometer-scale geological outcrop interpretation and further assess the robustness and generalization capability of the proposed framework.

With these improvements, the proposed framework is expected to evolve from a single segmentation and attitude-measurement tool into an intelligent interpretation method capable of handling multi-scale, multi-lithological, and cross-regional sedimentary outcrop point clouds, thereby providing a reliable data foundation and technical support for 3D geological modeling, sedimentary-unit analysis, and reservoir geometry characterization.

## Figures and Tables

**Table 1:** Ablation study results (%).

No.	Baseline	Geology-Prior-Enhanced Features	Weighted Random Sampling	CSA Module	Hybrid Loss Function	mIoU	OA	IoU-Outcrop
1	√					82.91	89.50	75.69
2	√	√				84.47	90.82	78.63
3	√	√	√			85.60	92.16	82.55
4	√	√	√	√		88.21	95.02	84.94
5	√	√	√	√	√	89.51	96.08	86.90

**Note:** “√” indicates that the corresponding module or strategy is included in the model.

**Table 2:** Performance comparison of different methods (%).

Method	mIoU	IoU-Outcrop	IoU-Vege	IoU-Soil	OA
DBSCAN	37.37	15.73	31.31	65.07	69.53
Random Forest	63.77	19.48	87.75	84.07	88.21
PointNet	38.86	25.81	28.10	62.66	66.11
PointNet++	82.91	75.69	85.58	87.45	89.50
SedCSA-Net	89.51	86.90	89.69	91.95	96.08

**Table 3:** Comparison of attitude measurements of fitted sedimentary layer boundaries.

Attitude Measurement Point	Ground Truth		SedCSA-Net	
	Dip Direction	Dip Angle	Dip Direction	Dip Angle
1	21.23	32.37	19.54	31.74
2	12.46	29.73	11.67	30.34
3	23.6	31.94	21.75	30.64
4	22.67	33.56	24.77	33.1

## Data Availability

The data presented in this study are available upon request to the corresponding author. As the data form part of ongoing research, they cannot be made publicly accessible at present.
